# Supplementary material for: Organic Electronics Picks Up the Pace: Mask‐Less, Solution Processed Organic Transistors Operating at 160 MHz
Source: Adv Sci (Weinh). 2021 Jan 4;8(4):2001098. doi: 10.1002/advs.202001098 (PMC7887599; doi:10.1002/advs.202001098)
Supplement: Supplementary file 1 — Supporting Information [file ADVS-8-2001098-s001.pdf]

© 2020 Wiley-VCH GmbH

ADVANCED SCIENCE
Open Access

Supporting Information

for *Adv. Sci.,* DOI: 10.1002/advs.202001098

Organic Electronics Picks Up the Pace: Mask-Less, Solution Processed Organic Transistors Operating at 160 MHz

*Andrea Perinot, Michele Giorgio, Virgilio Mattoli, Dario Natali, Mario Caironi**

Supporting Information

Organic Electronics Picks Up the Pace: Mask-Less, Solution Processed Organic Transistors Operating at 160 MHz

*Andrea Perinot, Michele Giorgio, Virgilio Mattoli, Dario Natali, Mario Caironi**

Methods

Materials: Aluminum Nitride substrates were purchased from MARUWA CO. The Ag-nanoparticle ink used in laser sintering (NPS-L) was purchased from HARIMA. Dimethylamino(benzenethiol) was purchased from TCI Chemicals. P(NDI2OD-T2) was purchased from Polyera. Polystyrene (M_w = 2000000), poly(vinyl cinnamate) (M_n = 40000) and 1,11-Diazido-3,6,9-trioxaundecane were purchased from Sigma Aldrich. The epoxy resin was purchased from Robnor.

FET fabrication: On the AlN substrates we first defined, via conventional photolithography, a set of structures and calibration patterns for connecting our FETs to the high-frequency probes for S-parameter measurement. The details on the structures can be found in *Giorgio et al.*[1] Then, we coated the Ag-nanoparticle ink onto these substrates via spin-coating at 7000 rpm for 5 min. Then, we patterned the source and drain bottom electrodes through laser sintering using the setup and following the procedures illustrated in our previous work.[2] In this case, the incident laser power was 17.2 mW at a scanning speed of 0.05 mm s^{-1}. The unprocessed part of the ink was removed by thorough rinsing with o-xylene. Then, Ar-plasma is applied for 4 minutes at a power of 100 W, and the self-assembly of DABT on the silver electrodes is induced by dipping the samples in a solution of 17 µl DABT in 12 ml of isopropanol for 15 minutes. The samples are then rinsed with isopropanol. The semiconductor layer is then deposited via off-centered spin-coating[3] (in nitrogen atmosphere) of a 7 g/l solution of P(NDI2OD-T2) in toluene, at a speed of 1000 rpm for 30 s. The samples are then annealed at 100 °C for 15 minutes. After cooling, a 40-nm-thick layer of polystyrene, mixed

with 1,11-Diazido-3,6,9-trioxaundecane at a weight ratio of 10:1, is deposited via spin-coating at a speed of 1500 rpm for 5 minutes from a solution in n-butyl acetate at a concentration of 7.5 g/l. Then, we spin-coated a solution of 50 g/l poly(vinyl cinnamate) in cyclopentanone at a speed of 1500 rpm for 2 minutes, so to yield a 300-nm-thick layer, which is then cross-linked analogously to the underlying layer. We then patterned the gate electrodes via laser sintering with the same procedure as illustrated above, using an incident power in the range 4.9-5.3 mW and a scanning speed of 0.02 mm s^{-1}. Finally, we encapsulated the devices by spin-coating a 50 g/l solution of PMMA in o-xylene at a speed of 1300 rpm for 60 s, followed by annealing at 60 °C for 20 min for solvent removal, followed by deposition of a 1-um-thick layer of parylene via CVD, finally completed by drop-casting a bi-component epoxy resin. After 24 h, the samples are then annealed for 8 h in nitrogen atmosphere at 105 °C.

Measurement: The thickness of the laser-sintered electrodes and of the polymer layers were measured with an Alpha-Step IQ profilometer by KLA-Tencor. The DC measurements were performed in nitrogen atmosphere using a Keysight B1500A Semiconductor Parameter Analyzer. The AC measurement was performed in ambient atmosphere using a setup and calibration method already described previously.[1] The parasitism attributed to the measurement pads and interconnections has been removed by measuring an open structure with a geometry identical to the interconnections used for the transistor measurement.[1]

Supporting Figures

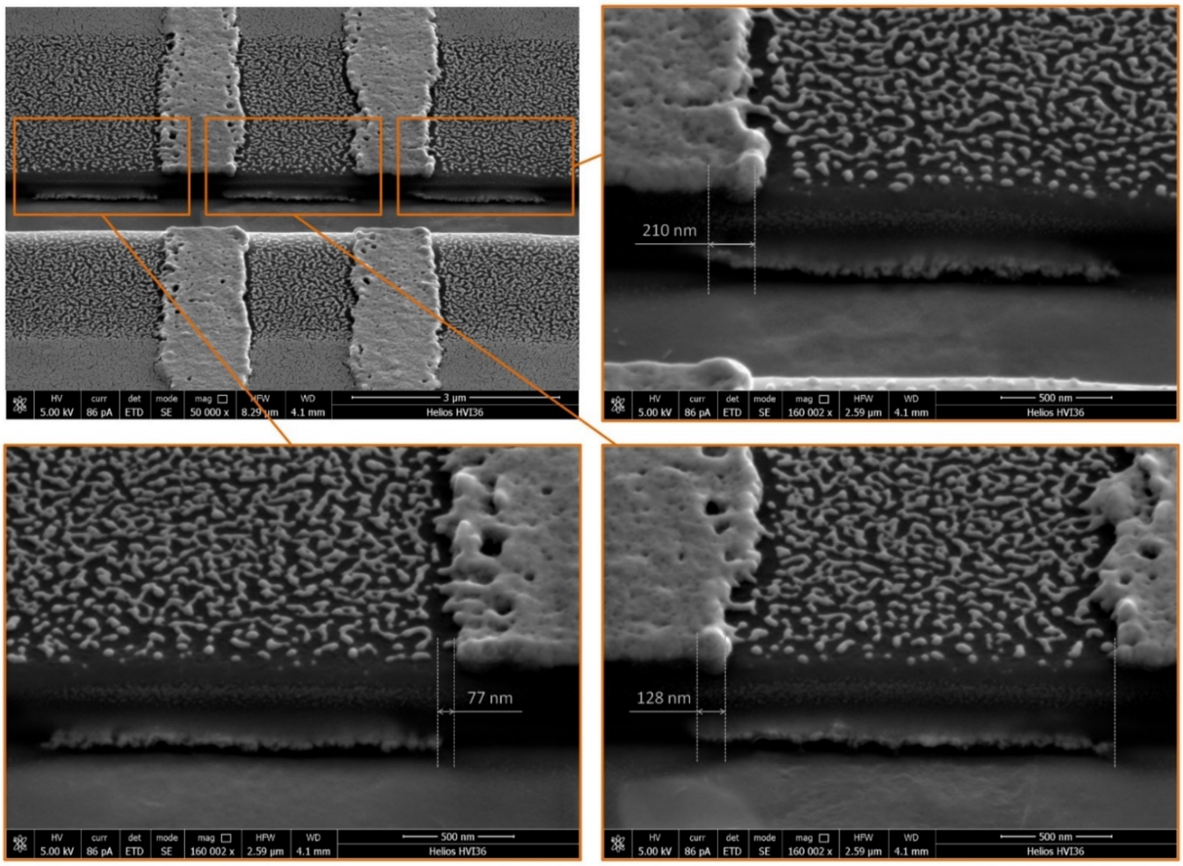

Figure S1: Cross-sectional SEM images of the realized device, with magnifications of the area in the vicinity of the bottom electrodes. Measurements of the electrode geometrical overlap are also shown.

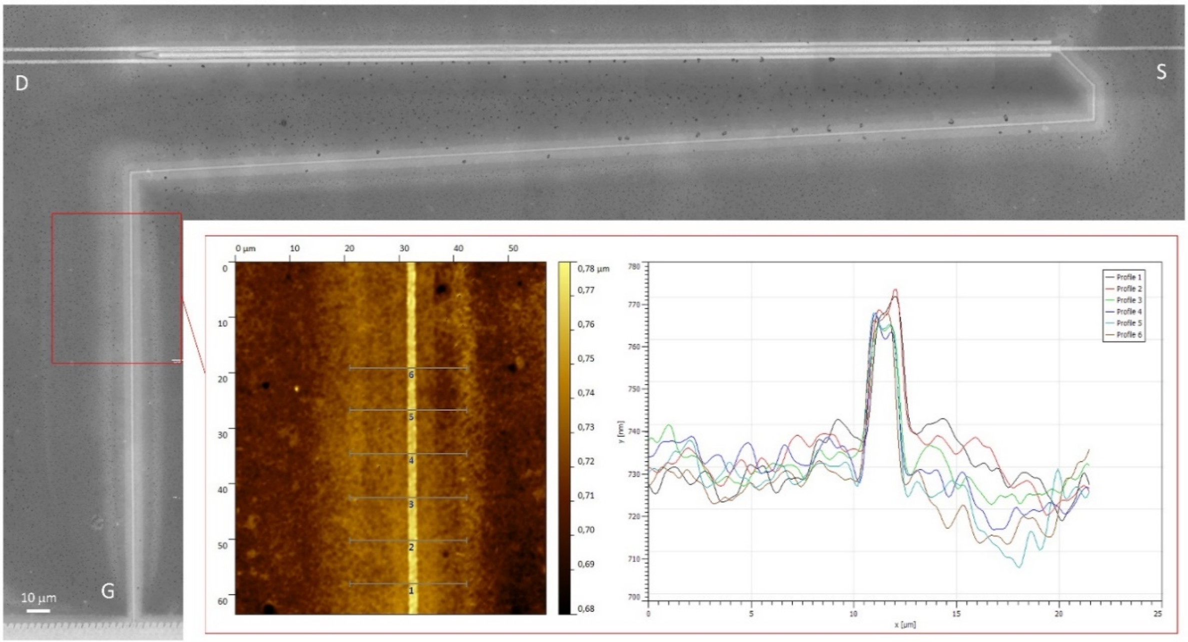

Figure S2: Optical image of a typical device and related confocal profilometry highlighting a particular of the laser-sintered gate track. The average thickness of the track on top of the dielectric stack is ~ 40-50 nm. All images were acquired with a Leica DCM 3D Confocal Profilometer, at 150x magnification. Profilometer data were elaborated with Gwyddion software (plane tilting, profile extraction, file conversion).

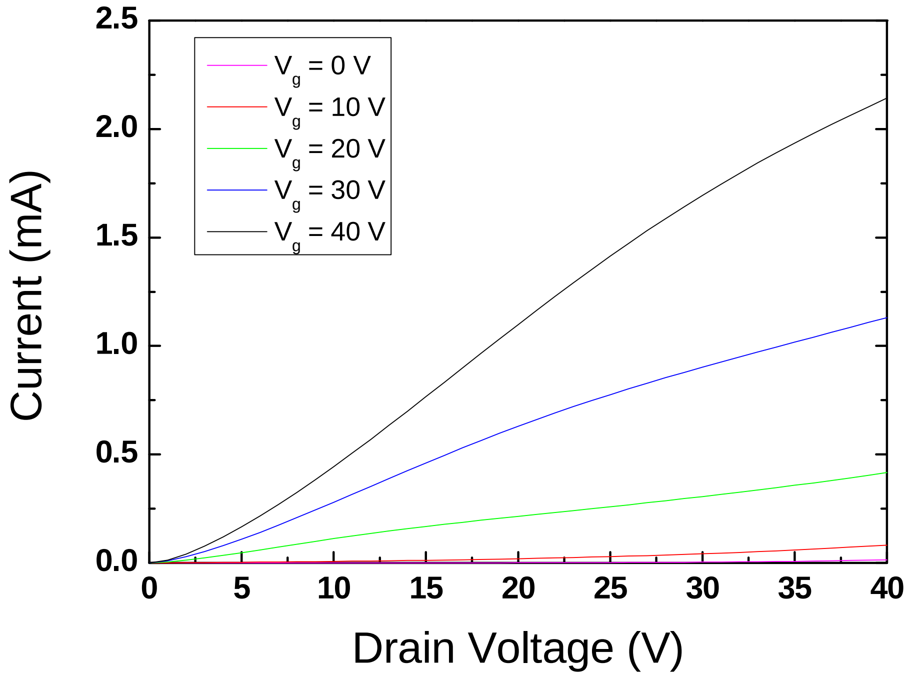

Figure S3: Measured output curve for the realized high-frequency OFET.

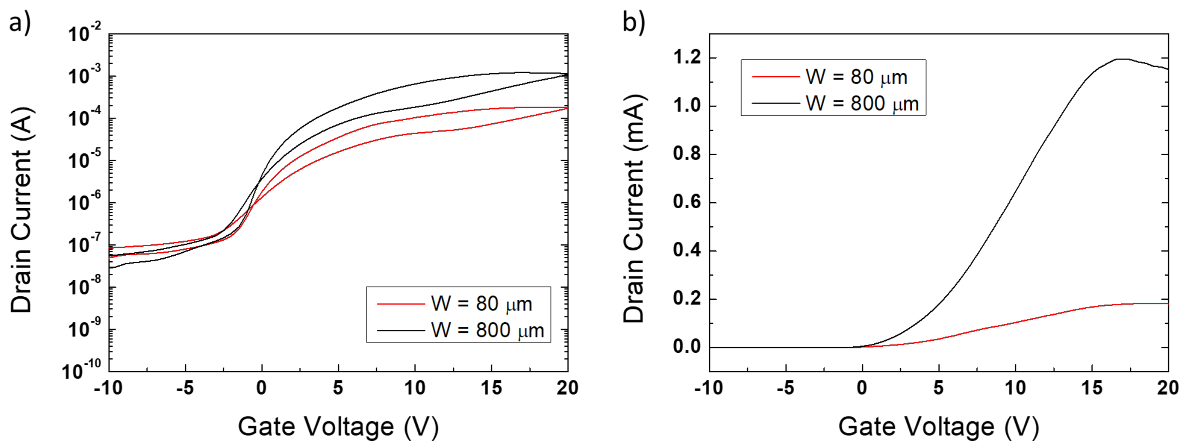

Figure S4: Measured transfer curves for OFETs on a glass substrate with low thermal conductivity, in the order of 1 W/mK. The devices are fabricated with the same architecture and comparable process as the ones fabricated on AlN substrate, and differ in terms of channel length and dielectric material (in this case, L = 1.4 µm, poly(vinyl alcohol) is used in place of poly(vinyl cinnamate) and the channel width is W = 800 µm or W = 80 µm). a) Transfer curves for V_d = 20 V in logarithmic scale. b) Same transfer curves (only forward scan) in the linear regime.

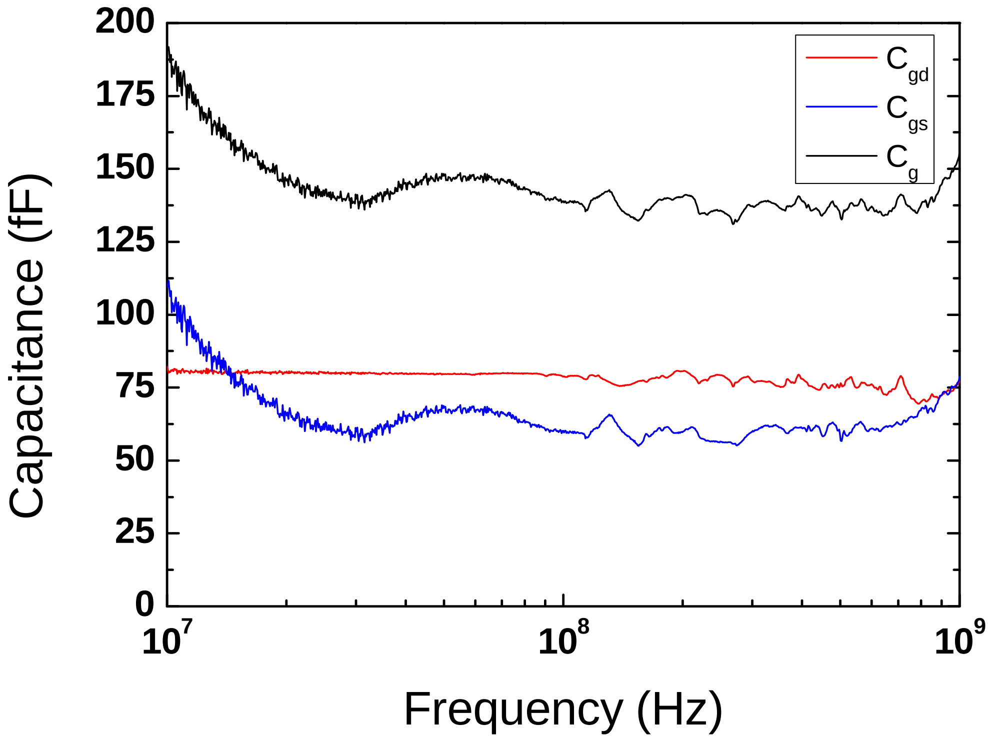

Figure S5: Calculated gate capacitances, extracted from the S-parameter measurement.

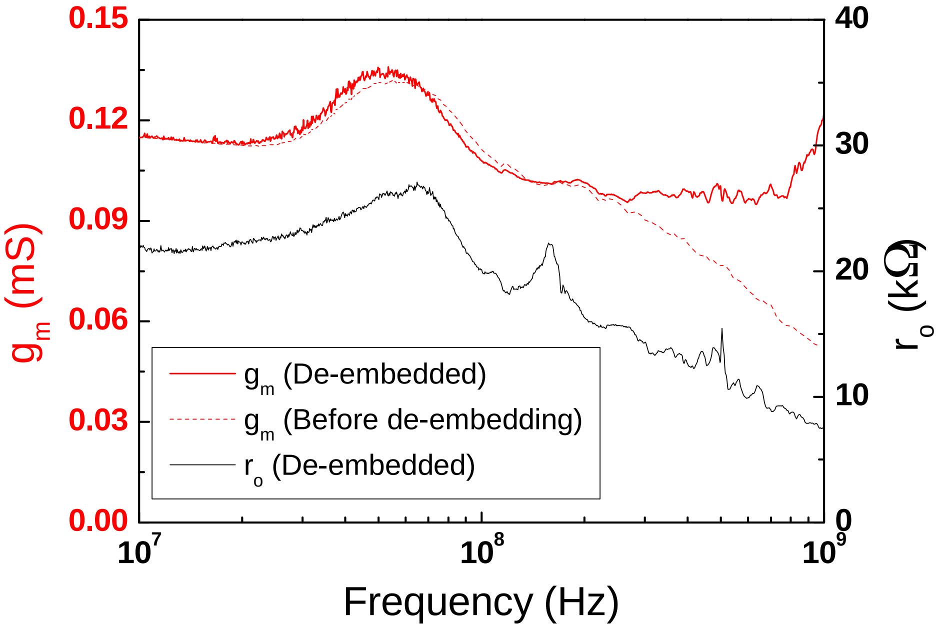

Figure S6: Calculated g_m and r_o, extracted from the S-parameter measurement. We show the extracted value for g_m both before and after the de-embedding.

Table S1: Selected results in the literature for high-frequency organic transistors and circuits, ordered in terms of f_t/V. Here reported only the works exhibiting f_t/V in excess of 1 MHz/V, in continuous-mode operation.

Reference	f_T (MHz)	f_t/V (MHz/V)[a)]	Flexible Substrate	Mask-less fabrication
This Work	160	4	X	Yes
Borchert et al.[4]	6.7	2.23	Yes	X
Perinot et al.[2]	14.4	2.06	Yes	Yes
Yamamura et al.[5]	20	2	X	X
Nakayama et al.[6]	19	1.9	X	X
Uno et al.[7]	25	1.67	X	X
Giorgio et al.[1]	19	1.27	X	Yes
Kitamura et al.[8]	27.7	1.11	X	X
Uemura et al.[9]	20	1	X	X

a) Our calculation when not reported. f_t is normalized to the highest voltage between source-gate or drain-gate. X: not applicable.

Extraction of the contact resistance

In the saturation regime, which is the case of interest here, only the contact resistance at source side matters (provided that voltage drop on the contact resistance at drain side is low enough to maintain the transistor in saturation[10]).

In the framework of the current crowding model, suitable for staggered transistors, contact resistances can be expressed as:

$$R_C = \frac{R_y}{W L_0 \tanh\left(\frac{L_{ov}}{L_0}\right)}, \tag{1}$$

Where: L_{ov} is the gate-contact overlap; R_y is the resistance per unit area taking into account injection and transport across the bulk; $L_0 = \sqrt{R_y / R_{sh}}$ is the injection length, viz. the characteristic length over which injection would take place for very large L_{ov}, R_{sh} being the channel sheet resistance. Modelling the carrier mobility as a power law, $\mu = \mu_0 \left(V_G - V_T\right)^{\gamma}$, the sheet resistance can be expressed as $R_{sh} = \left[\mu_0 C_{ins} \left(V_G - V_T\right)^{\gamma+1}\right]^{-1}$.

For the case of very small L_{ov}, which is the case of interest here (actually for $L_{ov} < L_0$, to be verified *a posteriori*), Equation (1) can be simplified as the sum of a constant term and of a V_G-dependent term, as it follows[11]:

$$R_C = \frac{R_y}{W L_{ov}} + \frac{1}{3} \frac{L_{ov}}{W} R_{sh} = R_{c,const} + R_{c,var}\left(V_G\right), \tag{2}$$

where the first term accounts for injection and transport across the film, whereas the second term accounts for transport along the film at the semiconductor/insulator interface.

The challenge in the saturation regime is due to the fact that the current voltage relationship incorporating the effects of contact resistance is actually an implicit function, without the possibility of writing current as an explicit function of V_G in the general case:

$$I = \frac{1}{\gamma+2} \mu_0 C_{ins} \frac{W}{L} \left(V_G - V_T - R_C I\right)^{\gamma+2}, \tag{3}$$

where V_T is the threshold voltage. There are 5 unknowns in Equation (3): μ_0, γ, V_T, $R_{c, const}$, L_{ov}, ($R_{c, var}$ can be expressed as a function of μ_0, γ, V_T, L_{ov}). To extract them from experimental data, we devise an iterative fitting algorithm. In addition, to ease the procedure and reduce the number of fitting parameters, we select reasonable ranges for μ_0 and V_T, and for each (μ_0 , V_T) couple we run the following algorithm.

The parameter γ is initialized at 0.01.

1. Since μ_0 and V_T are fixed and γ is initialized (or fitted, *vide infra*), we can calculate $\widehat{V_G}$, the base which is raised to (γ + 2) in Equation (3):

$$\widehat{V_G}=V_G-V_T-R_C I=\left(\frac{I(\gamma+2)}{\mu_0 C_{ins}\frac{W}{L}}\right)^{\frac{1}{\gamma+2}}. \tag{4}$$

2. Now we take advantage of the fact that: V_G and I are experimentally measured; μ_0 and V_T are fixed. We plot $V_G-V_T-R_C I$ versus V_G and, exploiting Equation (2), we fit $R_{c,const}$, L_{ov} and γ, with the constraint $\gamma > 0$. The fitting is done in the range 23 V < V_G < 40 V.
3. With the value for γ estimated at step 2, we jump to step 1 and reiterate for 100 cycles.

We sometimes experienced oscillations in the fitted value for γ between 0 and a certain $\tilde{\gamma}$. Indeed, for consistent and realistic fitted parameters, $\tilde{\gamma}$ is very close to 0 (actually smaller than 0.043), therefore the impact of such oscillation is negligible. In these cases, to proceed with the analysis, we arbitrarily chose $\gamma=\frac{\tilde{\gamma}}{2}$ and we run a final direct fit of R_c, determining $R_{c,const}$ and L_{OV}. Later, we verified that different choices for γ (*i.e.* $\gamma=\frac{\tilde{\gamma}}{4}$ or $\gamma=\frac{3}{4}\tilde{\gamma}$) did not appreciably change the results of the fitting.

The parameters γ, $R_{c,const}$ and L_{ov} extracted with μ_0 in the range 0.94 – 1.1 cm^2/Vs and V_T in the range 5.9-6.2 V are shown below in Table S2. From the independent measurement of the geometrical overlap between electrodes and of the dielectric thickness, within the framework

of the gate capacitance model illustrated in the main text,[12] we identify the acceptable values for L_{ov} (*i.e.* 0.34 µm < L_{ov} < 0.61 µm) and we highlight the corresponding combinations in red in Table S2.

Table S2: Extracted values of γ, $R_{c,const}$, L_{ov} as a result of the fitting of the experimental curves according to our algorithm. Values corresponding to the combinations where L_{ov} is within the acceptable range (according to a second independent measurement) are highlighted in red.

a) $R_{c,const}$ (Ω)

	Vt=5.9 V	Vt=5.92 V	Vt=5.94 V	Vt=5.96 V	Vt=5.98 V	Vt=6 V	Vt=6.02 V	Vt=6.04 V	Vt=6.06 V	Vt=6.08 V	Vt=6.1 V	Vt=6.12 V	Vt=6.14 V	Vt=6.16 V	Vt=6.18 V	Vt=6.2 V
mu0=0.94	7758	7952	8202	8540	9019	NA	NA	NA	NA	NA	NA	NA	NA	NA	NA	NA
mu0=0.96	6010	5990	5966	5938	5904	5861	NA	5738	5641	5498	5316	NA	NA	NA	NA	NA
mu0=0.98	5155	5086	5009	4920	4819	4702	4564	4399	4200	3952	3661	3248	2678	2629	2643	2657
mu0=1	4654	4574	4486	4389	4280	4158	4021	3865	3686	3478	3250	2960	2636	2650	2663	2677
mu0=1.02	4329	4248	4161	4066	3962	3848	3723	3584	3429	3256	3072	2850	2655	2669	2683	2697
mu0=1.04	4102	4025	3941	3852	3755	3651	3537	3414	3279	3134	2978	2797	2674	2688	2702	2716
mu0=1.06	3937	3864	3785	3701	3612	3516	3413	3303	3183	3059	2922	2770	2692	2707	2721	2735
mu0=1.08	3813	3743	3669	3591	3508	3420	3326	3226	3119	3010	2888	2756	2710	2724	2739	2753
mu0=1.1	3717	3650	3581	3508	3430	3349	3262	3171	3074	2977	2867	2750	2727	2742	2756	2771

b) L_{OV} (µm)

	Vt=5.9 V	Vt=5.92 V	Vt=5.94 V	Vt=5.96 V	Vt=5.98 V	Vt=6 V	Vt=6.02 V	Vt=6.04 V	Vt=6.06 V	Vt=6.08 V	Vt=6.1 V	Vt=6.12 V	Vt=6.14 V	Vt=6.16 V	Vt=6.18 V	Vt=6.2 V
mu0=0.94	13.1699	14.6752	16.9795	20.8868	28.5457	NA	NA	NA	NA	NA	NA	NA	NA	NA	NA	NA
mu0=0.96	4.8979	4.7725	4.6397	4.4983	4.3453	4.1732	NA	3.7606	3.4874	3.1401	2.7492	NA	NA	NA	NA	NA
mu0=0.98	3.0905	2.9158	2.7331	2.5414	2.3392	2.1243	1.8955	1.6509	1.3868	1.0996	0.8078	0.4627	0.0840	0.0349	0.0200	0.0051
mu0=1	2.3864	2.2271	2.0638	1.8960	1.7232	1.5450	1.3609	1.1704	0.9729	0.7678	0.5660	0.3436	0.1265	0.1111	0.0957	0.0804
mu0=1.02	2.0464	1.9037	1.7589	1.6119	1.4625	1.3106	1.1560	0.9985	0.8380	0.6744	0.5153	0.3443	0.2038	0.1880	0.1722	0.1564
mu0=1.04	1.8680	1.7383	1.6076	1.4758	1.3428	1.2087	1.0732	0.9365	0.7984	0.6609	0.5240	0.3812	0.2819	0.2656	0.2493	0.2332
mu0=1.06	1.7752	1.6552	1.5349	1.4141	1.2928	1.1711	1.0488	0.9260	0.8026	0.6821	0.5592	0.4338	0.3607	0.3439	0.3272	0.3106
mu0=1.08	1.7328	1.6203	1.5078	1.3952	1.2825	1.1697	1.0568	0.9438	0.8307	0.7216	0.6087	0.4950	0.4401	0.4229	0.4058	0.3887
mu0=1.1	1.7229	1.6162	1.5096	1.4032	1.2969	1.1908	1.0849	0.9791	0.8735	0.7722	0.6668	0.5614	0.5202	0.5026	0.4850	0.4675

c) γ

	Vt=5.9 V	Vt=5.92 V	Vt=5.94 V	Vt=5.96 V	Vt=5.98 V	Vt=6 V	Vt=6.02 V	Vt=6.04 V	Vt=6.06 V	Vt=6.08 V	Vt=6.1 V	Vt=6.12 V	Vt=6.14 V	Vt=6.16 V	Vt=6.18 V	Vt=6.2 V
mu0=0.94	6.8e-01	7.1e-01	7.6e-01	8.2e-01	9.2e-01	NA	NA	NA	NA	NA	NA	NA	NA	NA	NA	NA
mu0=0.96	4.0e-01	4.0e-01	4.0e-01	3.9e-01	3.9e-01	3.8e-01	NA	3.6e-01	3.5e-01	3.3e-01	3.0e-01	NA	NA	NA	NA	NA
mu0=0.98	2.9e-01	2.8e-01	2.7e-01	2.6e-01	2.5e-01	2.3e-01	2.1e-01	1.9e-01	1.7e-01	1.4e-01	1.1e-01	6.4e-02	6.1e-03	9.9e-05	9.9e-05	9.9e-05
mu0=1	2.3e-01	2.2e-01	2.1e-01	1.9e-01	1.8e-01	1.7e-01	1.5e-01	1.3e-01	1.1e-01	8.8e-02	6.3e-02	3.3e-02	9.9e-05	9.9e-05	9.9e-05	9.9e-05
mu0=1.02	1.9e-01	1.8e-01	1.7e-01	1.6e-01	1.4e-01	1.3e-01	1.1e-01	9.9e-02	8.2e-02	6.3e-02	4.3e-02	2.0e-02	9.9e-05	9.9e-05	9.9e-05	9.9e-05
mu0=1.04	1.6e-01	1.5e-01	1.4e-01	1.3e-01	1.2e-01	1.1e-01	9.3e-02	7.9e-02	6.5e-02	4.9e-02	3.2e-02	1.3e-02	9.9e-05	9.9e-05	9.9e-05	9.9e-05
mu0=1.06	1.4e-01	1.3e-01	1.2e-01	1.1e-01	1.0e-01	9.0e-02	7.9e-02	6.6e-02	5.3e-02	3.9e-02	2.5e-02	8.8e-03	9.9e-05	9.9e-05	9.9e-05	9.9e-05
mu0=1.08	1.3e-01	1.2e-01	1.1e-01	9.9e-02	8.9e-02	7.9e-02	6.8e-02	5.7e-02	4.5e-02	3.3e-02	2.0e-02	5.8e-03	9.9e-05	9.9e-05	9.9e-05	9.9e-05
mu0=1.1	1.1e-01	1.1e-01	9.7e-02	8.8e-02	7.9e-02	7.0e-02	6.0e-02	4.9e-02	3.9e-02	2.8e-02	1.6e-02	3.6e-03	9.9e-05	9.9e-05	9.9e-05	9.9e-05

In order to evaluate the goodness of the fitting resulting from the algorithm outlined above, we define as a figure of merit the quantity *err*, with the aim of weighting the goodness of fitting for both the current and the contact resistance:

1. We calculate the quantity $err_I = \sum_{V_g=23V}^{40V} \left(\frac{I - I_{fitted}}{I_{fitted}} \right)^2$

2. We calculate the quantity $err_{R_c} = \sum_{V_g=23V}^{40V} \left(\frac{R_c - R_{c,fitted}}{R_{c,fitted}} \right)^2$

3. We define $err = err_{R_c} + err_I$

The set of calculated quantities *err* for each combination of parameters μ_0 and V_t is presented In Table S3, where the acceptable values are highlighted in red with the same criterion as Table S2 above.

Table S3: Calculated values for *err* according to our algorithm. Values corresponding to the combinations where L_{ov} is within the acceptable range (according to a second independent measurement) are highlighted in red.

err

	Vt=5.9 V	Vt=5.92 V	Vt=5.94 V	Vt=5.96 V	Vt=5.98 V	Vt=6 V	Vt=6.02 V	Vt=6.04 V	Vt=6.06 V	Vt=6.08 V	Vt=6.1 V	Vt=6.12 V	Vt=6.14 V	Vt=6.16 V	Vt=6.18 V	Vt=6.2 V
mu0=0.94	0.05238	0.06414	0.08314	0.11798	0.19421	NA	NA	NA	NA	NA	NA	NA	NA	NA	NA	NA
mu0=0.96	0.00845	0.00833	0.00819	0.00801	0.00780	0.00752	NA	0.00674	0.00615	0.00537	0.00451	NA	NA	NA	NA	NA
mu0=0.98	0.00331	0.00312	0.00291	0.00269	0.00245	0.00221	0.00195	0.00169	0.00144	0.00120	0.00101	0.00091	0.00127	0.00139	0.00141	0.00143
mu0=1	0.00188	0.00176	0.00163	0.00150	0.00138	0.00125	0.00113	0.00102	0.00093	0.00086	0.00084	0.00092	0.00124	0.00126	0.00127	0.00129
mu0=1.02	0.00129	0.00122	0.00114	0.00107	0.00100	0.00093	0.00087	0.00082	0.00079	0.00078	0.00082	0.00093	0.00114	0.00115	0.00116	0.00118
mu0=1.04	0.00100	0.00095	0.00091	0.00086	0.00082	0.00078	0.00076	0.00074	0.00074	0.00076	0.00081	0.00093	0.00106	0.00107	0.00108	0.00109
mu0=1.06	0.00084	0.00081	0.00078	0.00075	0.00073	0.00071	0.00070	0.00070	0.00071	0.00074	0.00080	0.00091	0.00099	0.00099	0.00100	0.00101
mu0=1.08	0.00074	0.00072	0.00070	0.00068	0.00067	0.00066	0.00066	0.00067	0.00069	0.00073	0.00079	0.00088	0.00093	0.00094	0.00094	0.00095
mu0=1.1	0.00067	0.00066	0.00065	0.00064	0.00064	0.00064	0.00064	0.00066	0.00068	0.00072	0.00077	0.00085	0.00088	0.00088	0.00089	0.00090

The best fittings of the experimental data curves when combined with the constraints on the acceptable range of L_{ov} are identified for V_T = 6.1 V and 1.02 $cm^2/Vs < \mu_0 <$ 1.08 cm^2/Vs (Figure S7): indeed, the range for $\mu_0 \sim 1$ cm^2/Vs is consistent with independent reports for the adopted semiconducting polymer P(NDI2OD-T2)[10] and the range for V_T is reasonable and consistent with the measured transfer curves for our devices. In addition we verified that the injection length L_0 is larger than L_{ov}, as needed for equation (2) to hold (indeed Equation (2) is a very good approximation of Equation (1) already starting from $L_{ov} = L_0$, where the relative error is as low as 1.54%).[11]

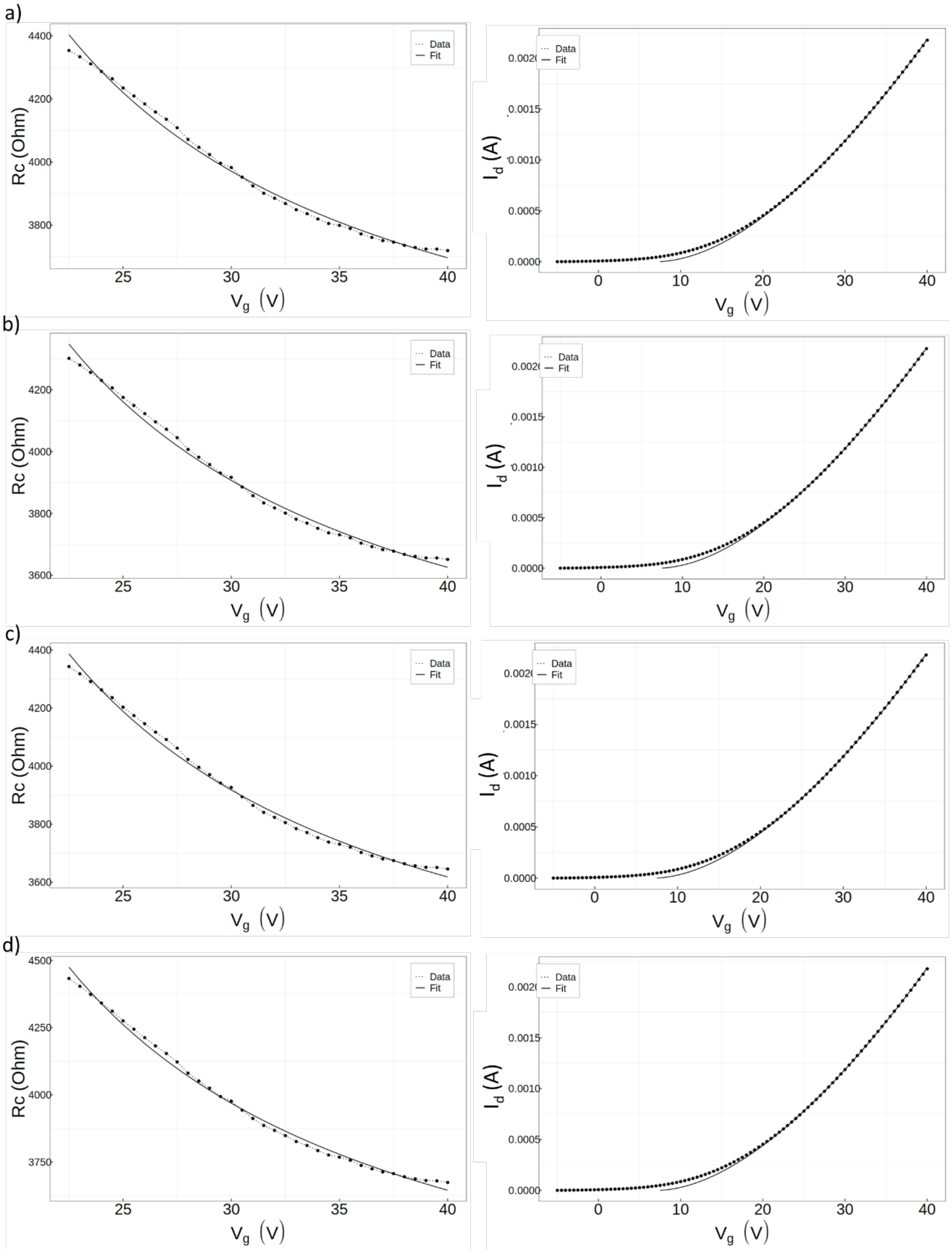

Figure S7: Experimental data and fitted curves as a result of our algorithm, for the combinations corresponding to V_T = 6.1 V and a) μ_0 = 1.02 cm^2/Vs, b) μ_0 = 1.04 cm^2/Vs, c) μ_0 = 1.06 cm^2/Vs, d) μ_0 = 1.08 cm^2/Vs.

For these ranges, 0.52 µm < L_{ov} < 0.61 µm, γ is approximately zero (below 0.043) and 2888 Ω < $R_{c,const}$ < 3072 Ω. Different choices for gamma returned extremely similar results:

- For $\gamma = \frac{1}{4}\tilde{\gamma}$: 0.54 μm < L_{ov} < 0.61 μm and 2912 Ω < $R_{c,const}$ < 3022 Ω,

- For $\gamma = \frac{3}{4}\tilde{\gamma}$: 0.41 μm < L_{ov} < 0.57 μm and 2768 Ω < $R_{c,const}$ < 3126 Ω.

In conclusion, we estimate for our high-frequency OFETs an R_c ~ 3600-3700 Ω at a bias voltage of 40 V. Such R_c is composed of a constant component estimated as $R_{c,const}$ ~ 3000 Ω and of a bias-dependent component calculated through Equation 2. The corresponding width-normalized contact resistance for our OFET is thus R_cW ~ 300 Ωcm at a bias of 40 V in the saturation regime.

Consistence of R_cW with the theoretical predictions for f_t

The experimental values reported here for f_t can be analyzed in the frame of a recently reported theoretical roadmap for high-frequency operation of organics. [12] With the model of that work, we express:

$$f_t = \frac{\mu_{eff}\left(V_G - V_T\right)}{2\pi L\left(\frac{2}{3}L + 2L_{ov}\right)}$$

where the parameters are defined analogously to the definitions in the main text, and

$$\mu_{eff} = \frac{\mu_0}{1 + \frac{\mu_0 R_c W}{L} C_{ins}\left(V_G - V_T\right)}$$

The contact resistance is described in accordance with the current-crowding model as in Equation (2), and considered as fully insisting on the source electrode.

When plugging in the parameters of the transistors of this work, as determined by the method described in the previous section, we obtain f_t ~ 138 - 146 MHz, which is consistent with the experimental measurement. We remark that, in the adopted model, the voltage dependence of the mobility on the gate voltage is not accounted for. However, such contribution is effective only at a second order, since $\gamma < 0.043$.

Bibliography

[1] M. Giorgio, M. Caironi, *IEEE Electron Device Lett.* **2019**, *40*, 953.
[2] A. Perinot, M. Caironi, *Advanced Science* **2019**, *6*, 1801566.
[3] N.-K. Kim, S.-Y. Jang, G. Pace, M. Caironi, W.-T. Park, D. Khim, J. Kim, D.-Y. Kim, Y.-Y. Noh, *Chem. Mater.* **2015**, *27*, 8345.
[4] J. W. Borchert, U. Zschieschang, F. Letzkus, M. Giorgio, M. Caironi, J. N. Burghartz, S. Ludwigs, H. Klauk, presented at *2018 IEEE International Electron Devices Meeting (IEDM)*, 1-5 Dec. 2018, 2018.
[5] A. Yamamura, S. Watanabe, M. Uno, M. Mitani, C. Mitsui, J. Tsurumi, N. Isahaya, Y. Kanaoka, T. Okamoto, J. Takeya, *Science Advances* **2018**, *4*.
[6] K. Nakayama, M. Uno, T. Uemura, N. Namba, Y. Kanaoka, T. Kato, M. Katayama, C. Mitsui, T. Okamoto, J. Takeya, *Adv Mater Interfaces* **2014**, *1*, 1300124.
[7] M. Uno, T. Uemura, Y. Kanaoka, Z. Chen, A. Facchetti, J. Takeya, *Org. Electron.* **2013**, *14*, 1656.
[8] M. Kitamura, Y. Arakawa, *Jpn. J. Appl. Phys.* **2011**, *50*, 01BC01.
[9] T. Uemura, T. Matsumoto, K. Miyake, M. Uno, S. Ohnishi, T. Kato, M. Katayama, S. Shinamura, M. Hamada, M.-J. Kang, K. Takimiya, C. Mitsui, T. Okamoto, J. Takeya, *Adv. Mater.* **2014**, *26*, 2983.
[10] D. Natali, L. Fumagalli, M. Sampietro, *J. Appl. Phys.* **2007**, *101*, 014501.
[11] D. Natali, J. Chen, F. Maddalena, F. García Ferré, F. Di Fonzo, M. Caironi, *Adv Electron Mater* **2016**, *2*, 1600097.
[12] A. Perinot, B. Passarella, M. Giorgio, M. Caironi, *Adv. Funct. Mater.* **2019**, *n/a*, 1907641.
